# Perception and decision mechanisms involved in average estimation of spatiotemporal ensembles

**DOI:** 10.1038/s41598-020-58112-5

**Published:** 2020-01-28

**Authors:** Ryuto Yashiro, Hiromi Sato, Takumi Oide, Isamu Motoyoshi

**Affiliations:** 1 Department of Life Sciences, The University of Tokyo, 3-8-1 Komaba, Meguro-ku, Tokyo 153-8902 Japan; 2 Department of Integrated Sciences, The University of Tokyo, 3-8-1 Komaba, Meguro-ku, Tokyo 153-8902 Japan

**Keywords:** Visual system, Human behaviour

## Abstract

A number of studies on texture and ensemble perception have shown that humans can immediately estimate the average of spatially distributed visual information. The present study characterized mechanisms involved in estimating averages for information distributed over both space and time. Observers viewed a rapid sequence of texture patterns in which elements’ orientation were determined by dynamic Gaussian noise with variable spatial and temporal standard deviations (SDs). We found that discrimination thresholds increased beyond a certain spatial SD if temporal SD was small, but if temporal SD was large, thresholds remained nearly constant regardless of spatial SD. These data are at odds with predictions that threshold is uniquely determined by spatiotemporal SD. Moreover, a reverse correlation analysis revealed that observers judged the spatiotemporal average orientation largely depending on the spatial average orientation over the last few frames of the texture sequence – a recency effect widely observed in studies of perceptual decision making. Results are consistent with the notion that the visual system rapidly computes spatial ensembles and adaptively accumulates information over time to make a decision on spatiotemporal average. A simple computational model based on this notion successfully replicated observed data.

## Introduction

Humans achieve stable perception of scenes and objects at a glance in spite of the spatial complexity and uncertainty of the natural image. While such perception seems to involve highly complicated and specialized neural processing, recent research has shown that perception builds upon image statistics computed relatively easily in the early stages of visual processing^1–4^. A vast psychophysical literature has suggested that the visual system is capable of rapidly estimating the characteristics of an ensemble of complex elements (e.g., objects, faces)^5–8^ as well as discriminating textures defined by simple visual features such as form, color and motion^9–16^. These studies offer clear evidence that the visual system automatically extracts a statistical representation of the spatial properties of the image. Such statistical visual representations are thought to be subserved by neural mechanisms in early visual cortex with large spatial receptive field or cortico-cortical interactions^17–23^.

Visual inputs inherently contain much temporal uncertainty owing to gaze shifts and object motions, and little is known about how mechanisms extracting spatial statistics cope with such temporal uncertainty. Psychophysical studies have examined how performance for orientation discrimination or global form detection in dynamic texture patterns varies as a function of stimulus duration. Results revealed that temporal summation is relatively short (over a few hundred milliseconds) and consistent with the idea that spatial statistics are computed rapidly by low-level mechanisms^24,25^. However, experiments using stochastic motion stimuli have shown that detecting global and biological motion requires a much longer temporal summation period (~10 sec)^26–28^ that is indicative of spatial mechanism with long time integration constants.

Recent psychophysical and physiological studies have conducted experiments in which observers estimate temporal statistics of noisy visual stimuli^28–34^. These studies introduce the idea that temporal integration is determined by a more dynamic process involving perceptual decision making^31,35^. Typical results obtained in these tasks (e.g., skewed distribution of reaction time, tradeoff between speed and accuracy) can be accounted for by a perceptual decision mechanism that accumulates sensory evidence over time toward a decision bound^36^. However, it remains unclear how such temporal integration interacts with the computation of spatial statistics.

The present study investigated associations between two seemingly distinct processes – computing spatial statistics and decision making – with a psychophysical paradigm in which observers estimated spatiotemporal stimulus statistics. In our study, human observers were exposed to a series of noisy textures and asked to discriminate average orientation over space and time. From the perspective of the classical spatial-averaging literature, one might expect that discrimination performance would be determined simply by the amount of spatiotemporal noise integrated by large receptive fields extending over both time and space. What we found instead, however, is that spatial and temporal noise have interactive effects on performance and that observers emphasize recent information in their judgments. We account for these data using a simple decision model based on dynamic accumulation of averaged spatial information. Our results raise the possibility that the visual system estimates spatiotemporal statistics of uncertain stimuli by rapidly computing spatial statistics and integrating them over time.

## Methods

### Observers

Six observers, four naïve and two of the authors (average age: 23.7) with corrected-to-normal vision, participated in the experiment. All experiments were conducted with a permission from the Ethics Committee of the University of Tokyo with written informed consent, and followed the Declaration of Helsinki guidelines.

### Apparatus

Visual stimuli were generated by a graphics card controlled by a PC and displayed on a LCD monitor (BenQ XL2730Z) which had a pixel resolution of 0.027 deg/pixel at a viewing distance of 50 cm we used. The refresh rate was 60 Hz. The mean luminance of the uniform background was 69.0 cd/m^2^. All experiments were conducted in a dark room.

### Stimuli

**Figure 1:**
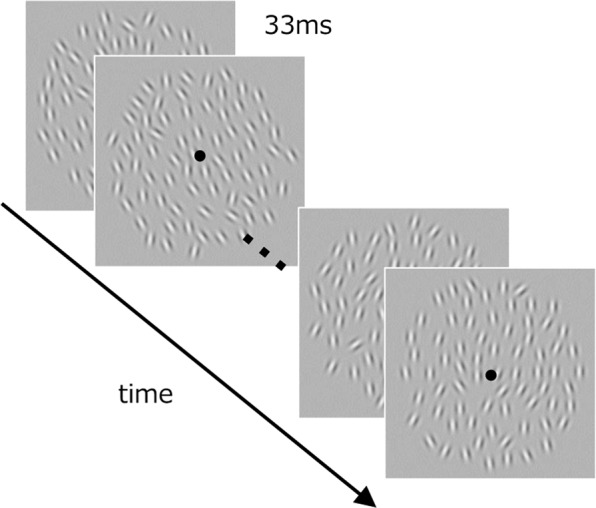
Schematic of a dynamic texture stimulus used in the experiment.

Stimuli were dynamic texture pattern which consisted of 4 or 32 frames presented one after the other in the center of the screen (Fig. 1). Each frame was presented for 33 ms (i.e., 30 Hz frame rate), which result in a total of 133 ms in 4 frames condition, and 1067 ms in 32 frames condition. The texture on each frame was composed of 70 Gabor patches, each of which was randomly placed with a minimum center-to-center separation of at least 0.86 deg from any other. The diameter of the texture was 10.7 deg. Each Gabor element had a carrier spatial frequency of 2.3 c/deg and a Gaussian window with a SD of 0.21 deg. The Michelson contrast was 0.8. The orientation of each Gabor element within a single texture frame was determined according to a normal distribution with spatial standard deviation of 0, 4, 8, or 16 deg (spatial SD: *σ*_*s*_). The spatial mean orientation of each texture frame was determined according to a normal distribution with a specific spatiotemporal mean (μ_st_) and temporal standard deviation of 0, 4, or 8 deg (temporal SD: *σ*_*t*_). For example, when [*μ*_*st*_, *σ*_*s*_, *σ*_*t*_] was [1, 16, 8 deg], the spatial mean orientation of each texture frame was set according to a normal distribution with a mean of 1 deg and SD of 8 deg, and the orientation of each element within each texture frame was set according to the normal distribution with that mean and SD of 16 deg.

### Procedure

For each condition, we measured discrimination thresholds for the spatiotemporal average orientation with a two-alternative forced choice procedure. On each trial, observers viewed the dynamic texture binocularly and indicated whether the spatiotemporal average of the orientation was tilted left or right by button press. Observers were instructed to respond within 1 sec after the stimulus offset. No feedback was given. The absolute spatiotemporal average of the orientation (|μ_*st*_|) was varied in accordance with the staircase method: it was decreased by 0.5 deg if the observer gave correct answers twice successively and it was increased by 0.5 deg if the observer gave an incorrect answer. In conditions where temporal SD was 0 deg, the amount of orientation change was set to 0.25 deg. The next trial started no less than 0.5 sec after the observer’s response. In each measurement session, multiple staircases corresponding to different conditions were randomly interleaved until the number of trials exceeded 30. Sessions were repeated until at least 180 trials (208.7 trials on average, 277 trials maximum) were collected for each condition. For each observer and condition, discrimination threshold was estimated by means of the maximum likelihood method. We did not consider the sign of observers’ responses in our analysis. This might lead us to conflate response-bias with sensitivity, but we confirmed that all observers were not significantly biased toward left or right across almost all the experimental conditions.

## Results

**Figure 2:**
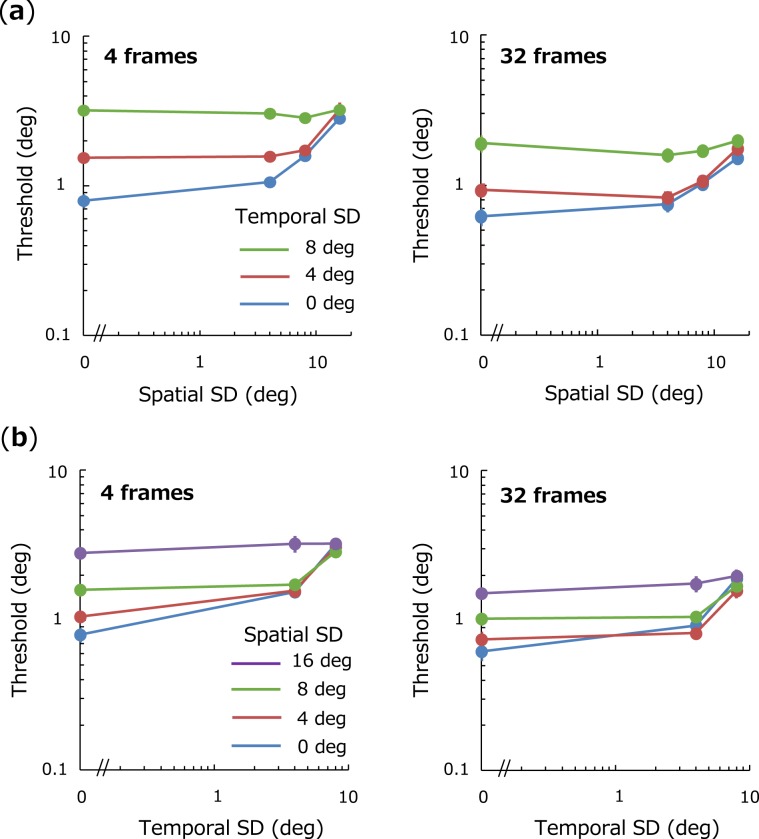
Effects of spatial SD and temporal SD on thresholds for spatiotemporal average. (**a**) Thresholds for spatiotemporal average-orientation as a function of spatial SD. Each panel shows thresholds for different number of frames (4 and 32, respectively). Different colors represent the results for different temporal SD. Error bars represent + −1 SE across observers. (**b**) Thresholds as a function of temporal SD. Different colors represent different spatial SD. Other conventions are the same as in (**a**).

Figure 2a shows discrimination thresholds for spatiotemporal average orientation as a function of spatial SD. Each color represents temporal SD, and each panel shows results for each total stimulus duration. Figure 2b shows a replot of the data as a function of temporal SD. We found that if temporal SD is small, discrimination thresholds increase as a function of spatial SD as has often been reported in previous studies: Threshold-vs-Noise (TvN) function^13,37^. If temporal SD is large, however, thresholds remain nearly constant. We conducted a three-way repeated measure ANOVA with factors of the number of frame, spatial SD, and temporal SD, and observed significant main effects of the number of frames (*F*(1,5) = 128, *p* < 0.001), spatial SD (*F*(3,15) = 19.4, *p* < 0.001), temporal SD (*F*(2,10) = 55.9, *p* < 0.001), and a significant interaction between spatial SD and temporal SD (*F*(6,30) = 14.7, *p* < 0.001). A multiple-comparison Shaffer test revealed that thresholds were significantly higher as spatial SD increases if temporal SD = 0 deg (*F*(3,15) = 65.6, *p* < 0.001) and temporal SD = 4 deg (*F*(3,15) = 18.6, *p* < 0.001), but not if temporal SD = 8 deg (*F*(3,15) = 1.43, *p* = 0.27). Results suggest that discrimination of spatiotemporal statistics is greatly influenced by spatial irregularity for temporally coherent streams but not for streams that fluctuate over time.

According to previous studies, observers’ discrimination thresholds can be assumed to be determined by a combination of internal and external noise (${\rm{Threshold}}=k\sqrt{{\sigma }_{int}^{2}+{\sigma }_{s}^{2}}$)^13,38,39^, where *σ*_int_ is internal noise, *σ*_s_ is spatial SD, and *k* is a scaling factor. We fit this model to observers’ thresholds for each temporal SD with *σ*_int_ and *k* being free parameters. We obtained best-fitting parameters as follows: for 32 frames condition, (*σ*_int_, *k*) = (4.82, 0.17), (8.07, 0.17), (52.4, 0.06) for temporal SD of 0, 4, 8 deg, respectively, and for 4 frames condition, (*σ*_int_, *k*) = (7.52, 0.09), (8.71, 0.09), (29.6, 0.06) for temporal SD of 0, 4, 8 deg, respectively.

## Discussion

To understand mechanisms underlying average orientation estimation for dynamic visual stimuli that fluctuate over space and time, the present study investigated thresholds for discriminating spatiotemporal average-orientation in successively presented noisy textures. We found an interaction between spatial and temporal noisiness: for stimuli containing less temporal fluctuation (i.e., smaller temporal SD), thresholds increased in a manner proportional to spatial noise whereas for stimuli with larger temporal fluctuation (i.e., larger temporal SD), they remained nearly flat regardless of the amount of spatial noise, thereby resulting in almost the same thresholds across temporal SD with largest spatial SD (=16 deg). These observed relationships are not surprising given that an incremental effect should be smaller as overall variance gets larger, but thresholds for large temporal SDs appear too flat against spatial SD.

What are the computational mechanisms that underlie our behavioral results? We first hypothesized that the observed tendencies could be explained by applying an extension of a spatial vision model^13,38^ for spatiotemporal average estimation. Specifically, it could be simply assumed that observers judged spatiotemporal average within a receptive field that integrates the whole number of elements equally over space and time. In this case, the observers’ performance is dependent on spatiotemporal SD denoted by the Eq. (1).

1 $${{\rm{\sigma }}}_{st}=\sqrt{{\sigma }_{s}^{2}+{\sigma }_{t}^{2}}$$

Since the receptive field does not distinguish different spatial and temporal locations, the model behaves in the same way in conditions with different spatial and temporal SDs but identical spatiotemporal SDs (e.g., (*σ*_s_, *σ*_t_) = (4, 8), (8, 4)). To test the validity of this model, we examined the relationship between spatiotemporal SD and discrimination thresholds.

**Figure 3:**
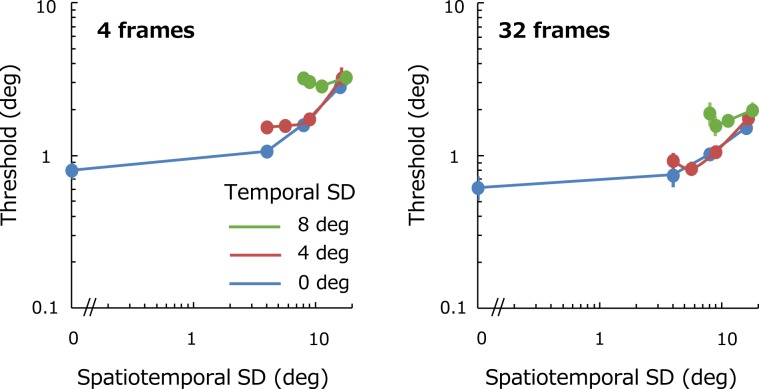
Discrimination thresholds as a function of spatiotemporal SD. Each panel shows thresholds for different number of frames (4 and 32, respectively). Different colors represent the results for different temporal SD. Error bars represent + −1 SE across observers.

Figure 3 is a replot of Fig. 2a,b as a function of spatiotemporal SD. Different curves represent thresholds for different temporal SDs. One possible prediction arising from the above account is that thresholds should be determined by spatiotemporal SD – in other words, all data points representing thresholds would be precisely on the same curve regardless of spatial and temporal SD. However, no systematic relationship was observed for both duration conditions. The results therefore do not support the extreme notion that observers integrate a series of orientations equally over space and time to estimate the average.

Considering the limited temporal resolution of local orientation coding in the visual system^15,40,41^, however, it is possible that the sensitivity of such a spatiotemporal receptive field would depend on the temporal variability of stimuli which in turn would result in lower absolute sensitivities for stimuli with large temporal SDs. This is consistent with larger internal noise obtained for large temporal SD conditions as described in the Results section. This spatiotemporal receptive field model, which integrates temporally blurred local orientation signals, could predict the trend of results observed in Fig. 3.

An alternative hypothesis inspired by findings about perceptual decision making is that the visual system could compute spatial average promptly for each temporal frame and then estimate overall average across all temporal frames. This idea is consistent with psychophysical data that humans are capable of extracting ensembles with surprising speed and accuracy^13,42^ even if they are composed of higher-order visual features including facial expressions^7^ and head direction^37^. As for spatial average-orientation, Dakin (2001) used spatially distributed visual stimuli with durations of only 100 ms and obtained average-orientation discrimination thresholds similar to our results^13^. In addition, behavioral and physiological studies have unequivocally indicated general mechanisms (e.g., Drift Diffusion Model^43^) based on accumulation of sensory evidence toward a bound underlying human and primate perceptual decision making^44,45^. On the basis of these findings, it is also sensible to assume computational mechanisms whereby spatial average-orientation is rapidly estimated and continually integrated over time.

To arbitrate between these possible mechanisms, we turned to another aspect of human data: the temporal dynamics of integration processes. Importantly, a number of psychophysical studies suggest that humans tend to give more weight to later inputs than to earlier ones^29–31,33,34^. This is often called the recency effect. It is plausible that observers in our experiments did not weigh information evenly over time as well. To test if this is the case, we calculated logistic regression coefficients β of spatial average-orientation *θ*(t) upon the observer’s response.

2 $${\rm{logit}}\left(p\right)=\,\log \left(\frac{p}{1-p}\right)=\alpha +\beta \theta \left(t\right)$$

The coefficient β can be regarded as the “impact” of spatial average at each temporal frame on the observer’s estimation. Correspondingly, a higher impact for a particular frame means that an observer emphasizes information at that frame. The former linear spatiotemporal integration model essentially predicts flat impact curves whereas the latter model might predict curves with higher impacts around stimulus offset (i.e., recency effect).

**Figure 4:**
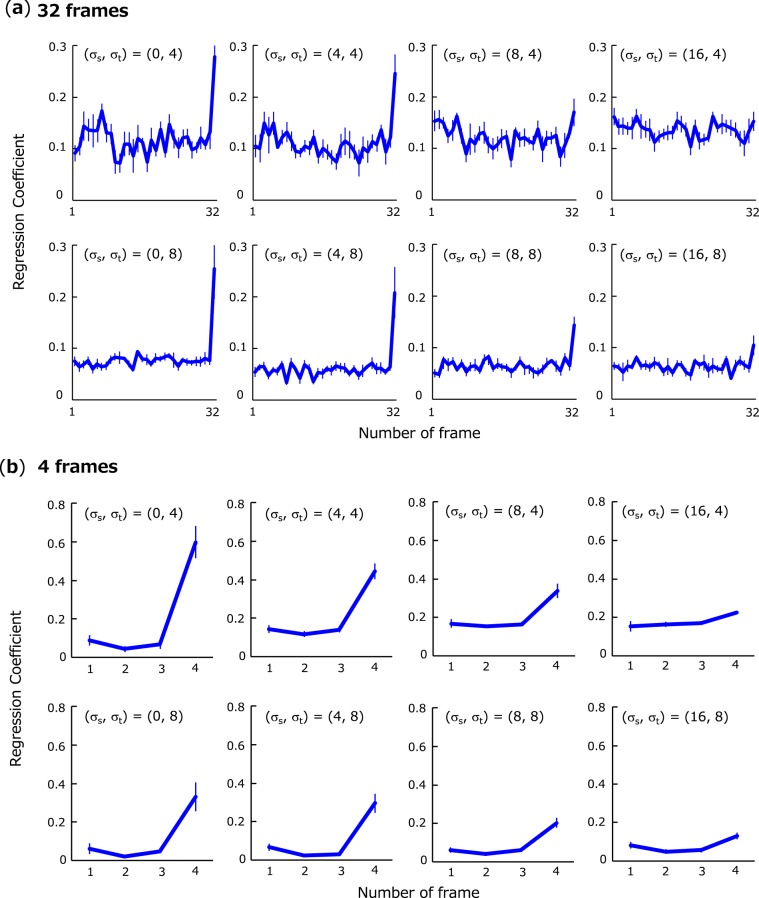
Impacts at each frame upon spatiotemporal average estimation. Error bars represent + −1 SE. The results are shown for 32 frames (**a**) and for 4 frames (**b**).

Figure 4 shows impacts obtained for various spatial and temporal SDs (*σ*_*s*_ and *σ*_*t*_) except for zero temporal SD. Figure 4a shows the results for exposure duration of 32 frames and Fig. 4b show the results for 4 frames. Consistent with a number of the previous results^29–31,33,34^, we observed a clear recency effect: in particular, the impact of the last frame is higher than those of earlier frames, but this effect becomes less marked as spatial SD becomes larger. A three-way repeated measure ANOVA on impacts with factors of spatial SD, temporal SD and temporal location (last frame vs. first to 31st frame) revealed a significant main effect of spatial SD (*F*(3,15) = 4.83, *p* = 0.02), temporal SD (*F*(1,5) = 10.0, *p* = 0.03), and temporal location (*F*(1,5) = 15.7, *p* = 0.01), and a significant interaction between spatial SD and temporal location (*F*(3,15) = 7.00, *p* = 0.004). A multiple-comparison test showed the last impact was significantly higher than those of other frames when spatial SD is less than 8 deg (*F*(1,5) > 9.6, *p* < 0.027), but not when spatial SD is 16 deg (*F*(1,5) = 3.16, *p* = 0.14), thereby indicating that observers estimate spatiotemporal average-orientation by focusing heavily on later inputs except for stimuli with too much spatial variability. Therefore, the results clearly rule out linear spatiotemporal integration mechanisms and support the spatial-average integration mechanisms.

### Computational model of spatiotemporal average discrimination

**Figure 5:**
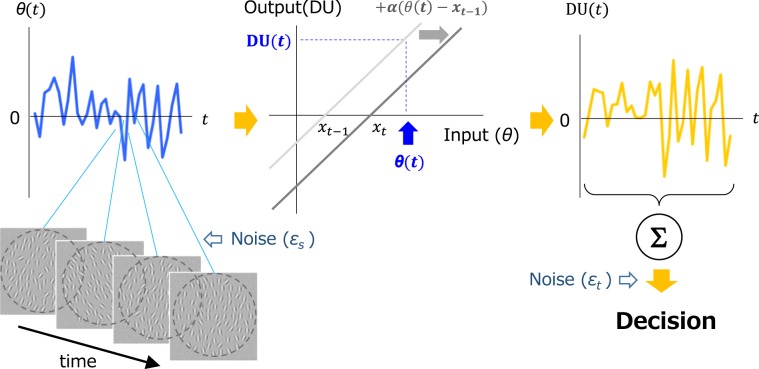
A computational model for spatiotemporal averaging. A large receptive field quickly estimates spatial average-orientation at each temporal frame. Each average is converted into a decision update (DU) by a linear transducer function that adaptively shifts over time. The final decision is based on the sum of DU throughout the presentation and additive internal noise.

On the basis of the above findings, we propose a distinct computational model that consists of the following processes: rapid spatial average estimation and a linear accumulation of sensory evidence (Fig. 5). In this model, spatial average-orientation is computed at each temporal frame by visual units with a large receptive field – an assumption corroborated by previous studies^13,15,38,46,47^ – and transferred to the evidence accumulation phase. Importantly, gain control modulates the accumulation: an input is converted into decision-relevant information (decision update; DU) by a linear transducer function that constantly shifts according to preceding inputs, and integrated over time to make a decision.

First, spatial average-orientation (*θ*(*t*)) at each temporal frame is estimated based on information within the large receptive field. According to previous studies, the visual system extracts a statistical summary by sampling a limited number of items rather than parallel processing across all items^48–50^: even if N elements are presented, the visual system in fact integrates only $\sqrt{N}$ elements^7,13,32,51^. In our experiment, 8 (≒ $\sqrt{70}$) elements are randomly chosen, and θ(*t*) is thus equivalent to the average orientation of these elements in addition to internal noise *ε*_*s*_.

Second, spatial average (*θ*(*t*)) is converted by a linear transducer function of an input into DU:

3 $${\rm{DU}}(t)=\theta (t)-{x}_{t-1}$$

*x*_*t*_, centroid of the function, is constantly updated according to the following equation.

4 $${x}_{t}={x}_{t-1}+\alpha (\theta (t)-{x}_{t-1})$$

The learning rate *α* determines the extent to which the function horizontally shifts. This shift makes the model adaptive, as an input is adjusted by preceding inputs.

The third and final decision stage is determined by the linear summation of DU over all temporal frames and additive internal noise *ε*_*t*_.

5 $$S=\mathop{\sum }\limits_{t=0}^{T}{\rm{DU}}(t)+{\varepsilon }_{t}$$

The model judges average orientation as tilted clockwise or counterclockwise, if *S* is positive or negative, respectively.

We simulated discrimination thresholds and impact curves using only three free optimized parameters (*α*, *ε*_s_, *ε*_t_). We searched for best-fitting parameters that minimized the chi-squared error between the predicted and observed data (thresholds of all the conditions and impacts of the conditions with non-zero temporal SDs). Since the number of data points for thresholds was smaller than that for impacts, we adjusted the error by multiplying a scaling factor so that thresholds and impacts made equal contributions to the fit. The model fitting was done independently for two different frame durations.

**Figure 6:**
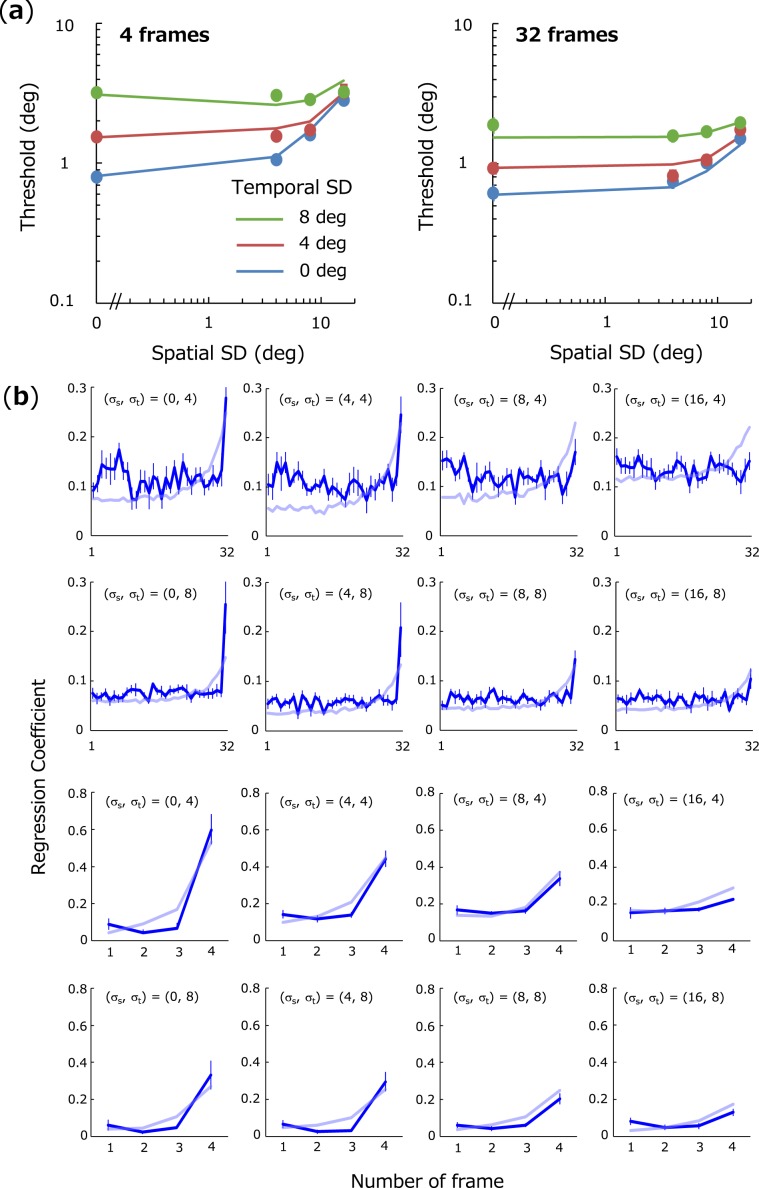
Simulation results by the hybrid model. (**a**) Discrimination thresholds as a function of spatial SD (solid lines). Each color corresponds to thresholds for each temporal SD. Filled circles represent human data. (**b**) Impact curves predicted by the model (faint blue lines) and human data (blue lines). Panels are arranged in the same fashion as Fig. 4.

We obtained the best fit when (*α, ε*_s_, *ε*_t_) = (0.18, 2.36, 2.97) for 32 frames (*χ*^2^ = 64.6), and (0.50, 1.04, 1.10) for 4 frames (*χ*^2^ = 18.0). Since the model generates values from probabilistic distributions (e.g., *θ*(*t*)), the best-fitting parameters vary slightly but not substantially every time data are simulated because of the large number of trials. Simulated results bear qualitative similarity to human data in terms of thresholds (Fig. 6a) and recency effects (Fig. 6b) although several mismatches are found in the absolute values of the estimated impacts and thresholds. We also simulated thresholds and impacts of alternative models such as the one that linearly integrates spatial average with *α* set to 0 (i.e., without gain control), and the one that responds to the average orientation of the last frame only. These models essentially failed to replicate human data. All evidence considered, the present results are consistent with a simple model of the visual system whereby spatial average signals accumulate over time as mediated by gain control to estimate spatiotemporal average-orientation of dynamic texture.

One central assumption of our model is gain control whereby the spatial average signal is constantly adjusted across temporal frames. Some previous studies also incorporated gain control in their models and successfully accounted for human tendencies^31,52^. Another central assumption is the limited sampling of $\sqrt{N}$ elements in estimating spatial average orientation for each frame. These distinct mechanisms should lead to the characteristic thresholds (Fig. 2). That is, if spatial SD is small, spatial average is accurately estimated in spite of the limited sampling and the linear transducer function is adaptively updated in a way that produces a DU nearly consistent with a correct response. In this particular case, performance depends only on temporal SD. By comparison, spatial average is poorly estimated due to limited sampling if spatial SD is large and the function shifts in a manner that produces a DU inconsistent with a correct response. This leads to poor performance in this case, regardless of temporal SD.

While the present model successfully replicates psychophysical data with few fixed parameters, the distinct mechanisms we have shown for spatial and temporal integration might be specific to our stimuli in which elements were temporally mixed due to the spatial overlap of elements across frames. We would have obtained different results and computational models, such as linear integration for both space and time, had we used stimuli without temporal mixture. To test if temporal mixture has an influence on our results, we used stimulus value from the experiment and generated effective orientations at each frame by averaging elements in neighboring two frames if the center distance of those elements was less than the Gaussian window SD of each Gabor element (0.21 deg), and then calculated the effective spatiotemporal SD caused by temporal mixture. We confirmed that spatiotemporal SD was reduced only by 5% in comparison with the original across all conditions. Although our way of calculating effective spatiotemporal SD is just one of the possible procedures, it is reasonable to conclude that temporal mixture is not likely to have a critical influence on our results, notwithstanding the possibility that our model is stimulus specific. Yet, given the limited temporal resolution of local orientation coding, it seems likely that spatial average orientation would be calculated on the basis of temporally blurred local orientation signals regardless of whether our stimuli contained temporal mixture. The present study thus leaves room for improvement in the model by incorporating such characteristics of the visual system.

As stated above, we observed some discrepancies between our data and the model: the recency effect almost totally disappears in human data at the largest spatial SD (16 deg) while the model still predicts it as a corollary of gain control. Since the model in fact predicts some decrease in the recency effect as spatial SD becomes larger, the inconsistency might be merely subject to the nonlinear scaling of spatial SD^15^. It is also possible that a distinct mechanism is involved, exceptionally, if stimuli are so spatially noisy that observers have difficulty in estimating average and/or cannot perceive the spatially-averaged orientation consciously^29^. In this case, mechanisms with spatiotemporal receptive fields (with a long time constant^27^) might be responsible for discrimination.

Another notable discrepancy is that the increase of impact near the last frame, which characterizes the recency effect, appears too steep in human data as compared to the model prediction. This might be partly owing to the visible persistence of the last frame^53^, backward masking of the previous frames by the last frame^54^, or temporal crowding, in rapid presentation^55,56^. However, it is doubtful that such perceptual effects can entirely explain our results because our subsequent experiments confirmed that such a steep recency effect is still robustly observed even if frame rate was reduced to as slow as 10 Hz and the average orientation of every frame (100 ms) was perceived very clearly. In addition, the extreme model whose decisions based only on the last frame was inconsistent with human behaviors in terms of the absolute values of thresholds and impacts. These facts do not seem to support backward masking as an explanation for the steep impact curves, but it is still possible that backward masking could influence the results and, if so, recency effects commonly observed in previous decision-making studies^29–31,33,34^ might be also attributed to backward masking rather than decision processes. Further work is yet required to establish the extent to which backward masking leads to biased temporal weight to successive visual stimuli during evidence accumulation.

Additionally, the neural implementation of our model deserves consideration. While parietal and frontal areas have been identified as a neural locus for temporal accumulation^57–59^, it remains unclear at which level neurons with the large receptive field for spatial average estimation exists in the visual system. One possibility is that V1 or V2 neurons for texture processing involve such spatial mechanisms, but they might not be able to capture all the elements that were widely distributed in our stimulus setting. Another possibility is feedforward and feedback connections in which neurons at an early visual area rapidly process each frame and receive recursive signals from higher levels in order to establish conscious ensemble perception^23,60^. This idea appears to be consistent with our assumption as neurons in V4 or MT have larger receptive fields. In view of this, the rapid stimulus presentation might impair the slow recursive signal, adding noise to observers’ perception of spatial average orientation. It seems unlikely that only low-level visual system is linked to successive spatial integration, although further experiment will be needed to delineate in more detail neural representation of the large receptive field.

The present study characterized human average estimation of spatiotemporally distributed visual information and suggests a simple model based on a rapid perceptual system followed by an integrative decision system. The experiments and analysis used herein provide a unified and wide-ranging paradigm to investigate perceptual mechanisms responsible for spatial and temporal ensemble statistics that have been studied independently (see Introduction). Future investigations may extend this approach to more systematic analyses across stimulus parameters and to a variety of visual attributes such as motion and faces. We expect that these investigations will update the current model which were developed with limited data and under a restricted set of conditions.

## Data Availability

The datasets generated and analyzed during the current study are available from the corresponding author upon reasonable request.
